# CRISPR for Crop Improvement: An Update Review

**DOI:** 10.3389/fpls.2018.00985

**Published:** 2018-07-17

**Authors:** Deepa Jaganathan, Karthikeyan Ramasamy, Gothandapani Sellamuthu, Shilpha Jayabalan, Gayatri Venkataraman

**Affiliations:** Plant Molecular Biology Laboratory, Department of Biotechnology, M. S. Swaminathan Research Foundation, Chennai, India

**Keywords:** CRISPR, TALEN, ZFN, quantitative trait loci, biotic stress, abiotic stress

## Abstract

The availability of genome sequences for several crops and advances in genome editing approaches has opened up possibilities to breed for almost any given desirable trait. Advancements in genome editing technologies such as zinc finger nucleases (ZFNs), transcription activator-like effector nucleases (TALENs) has made it possible for molecular biologists to more precisely target any gene of interest. However, these methodologies are expensive and time-consuming as they involve complicated steps that require protein engineering. Unlike first-generation genome editing tools, CRISPR/Cas9 genome editing involves simple designing and cloning methods, with the same Cas9 being potentially available for use with different guide RNAs targeting multiple sites in the genome. After proof-of-concept demonstrations in crop plants involving the primary CRISPR-Cas9 module, several modified Cas9 cassettes have been utilized in crop plants for improving target specificity and reducing off-target cleavage (e.g., Nmcas9, Sacas9, and Stcas9). Further, the availability of Cas9 enzymes from additional bacterial species has made available options to enhance specificity and efficiency of gene editing methodologies. This review summarizes the options available to plant biotechnologists to bring about crop improvement using CRISPR/Cas9 based genome editing tools and also presents studies where CRISPR/Cas9 has been used for enhancing biotic and abiotic stress tolerance. Application of these techniques will result in the development of non-genetically modified (Non-GMO) crops with the desired trait that can contribute to increased yield potential under biotic and abiotic stress conditions.

## Introduction

In the current scenario, the most critical challenge faced by the human race is to provide food security for a growing population. By 2050, the human population will reach 10 billion and to feed the world, global food production needs to increase by 60–100% (FAOSTAT, 2016). Besides the growing population rate, extreme weather, reduced agricultural land availability, increasing biotic and abiotic stresses are significant constraints for farming and food production. Development of technologies that can contribute to crop improvement can increase production to some extent. Genetic manipulation techniques using physical, chemical and biological (T-DNA insertion/transposons) mutagenesis have contributed majorly in studying the role of genes and identifying the biological mechanisms for the improvement of crop species in the past few decades (Ma et al., 2016). For the past three decades, transgenic techniques have been used to understand basic plant biology and also used for crop improvement. However, the integration of transgenes into the host genome is non-specific, sometimes unstable and is a matter of public concern when it comes to edible crop species (Stephens and Barakate, 2017).

In the last decade, the use of genome editing technologies with site-specific nucleases (SSNs) has successfully demonstrated precise gene editing in both animal and plant systems. These SSNs create double-stranded breaks (DSB) in the target DNA. The DSBs are repaired through non-homologous end joining (NHEJ) or homology-directed recombination (HDR) pathways resulting in insertion/deletion (INDELS) and substitution mutations in the target region(s), respectively (Jinek et al., 2012). In contrast to the transgenic approach, which leads to random insertions and very often random phenotypes, genome editing methods produce defined mutants, thus becoming a potent tool in functional genomics and crop breeding. Genome edited crops have an additional advantage over transgenic plants since they ‘carry’ their edited DNA for the desired trait (Malzahn et al., 2017). Such improved crops can be used in breeding programs and the resulting varieties can be used directly with lesser acceptability/consumption issues and relatively lesser regulatory procedures compared to conventional genetically modified (GM) crops (Waltz, 2018). This review discusses the advantages and applications of second-generation genome editing techniques such as CRISPR/Cas9 and its derivatives over the first-generation genome editing tools such as meganucleases, zinc finger nucleases (ZFNs) and transcription activator-like effector nucleases (TALENs).

## Engineered Nucleases – the New ERA of Genome Editing

Engineered nucleases contain a non-specific nuclease domain fused with a sequence-specific DNA binding domain. Such fused nucleases can precisely cleave the targeted gene and the breaks can be repaired through NHEJ or HDR and hence the term ‘genome editing’ (Gaj et al., 2013). First generation genome editing technologies that use meganucleases, ZFNs and TALENs involve tedious procedures to achieve target specificity, are labor intensive and time-consuming. In contrast, second-generation genome editing techniques including CRISPR/Cas9 involve easier design and execution methodologies that are also more time- and cost-effective. The review now briefly dwells on first generation genome editing tools and subsequently elaborates on second-generation genome editing tools with an emphasis on the use of CRISPR/Cas9 and its utilization to elucidate processes in plant biology. ZFNs are utilized widely for genome editing for more than a decade in both animal and plant systems (Govindan and Ramalingam, 2016). In the current scenario, ZFNs are less preferred due to their low target specificity, labor-intensive nature, many off-targets cleavages and a limited number of available target sites (Chen and Gao, 2013). As the mechanisms and applications of ZFNs, have been described in earlier reviews, we are not explaining in detail in this review (Kim et al., 1996; Gaj et al., 2013; Pauwels et al., 2014; Osakabe and Osakabe, 2015). TALENs, are engineered by modifying transcription activator-like effector (TALE) domain repeats for desirable target recognition and are subsequently fused with the FokI nuclease resulting in a TALEN suitable for target genome editing. Engineered TALENs recognize 18–20 bp stretches, similar to ZFNs, with a pair of TALENs required for FokI dimerization containing a spacer of 14-20 bp (Stephens and Barakate, 2017). TALENs show higher target binding specificity compared to ZFNs due to their length. However, with a requirement of a thymidine base at the starting position, large size and repetitive nature, TALENs are challenging to design and assemble. TALENs have been used for genome editing in plants including *Arabidopsis* (Cermak et al., 2011), rice (Li et al., 2012), tobacco (Zhang et al., 2013) and *Brachypodium* (Shan et al., 2013).

## Clustered Regularly Interspaced Palindromic Repeats (CRISPR/Cas9)

The discovery of CRISPR/Cas9 gene editing system has revolutionized research in animal and plant biology with its utility in genome editing being first demonstrated in 2012 in mammalian cells (Jinek et al., 2012). Unlike ZFNs and TALENs, CRISPR genome editing is more straightforward and involves designing a guide RNA (gRNA) of about 20 nucleotides complementary to the DNA stretch within the target gene. The acronym CRISPR, (first coined in 2002; Jansen et al., 2002) refers to tandem repeats flanked by non-repetitive DNA stretches that were first observed in the downstream of *Escherichia coli iap* genes (Ishino et al., 1987). In 2005, these non-repetitive sequences were found to be homologous with foreign DNA sequences derived from plasmids and phages. Subsequently, the mechanism of homology-dependent cleavage was explored for genome editing and the technology of CRISPR/Cas9 cleavage ‘arrived’ as a promising genome editing tool (Mojica et al., 2005; Liu et al., 2017).

**FIGURE 1:**
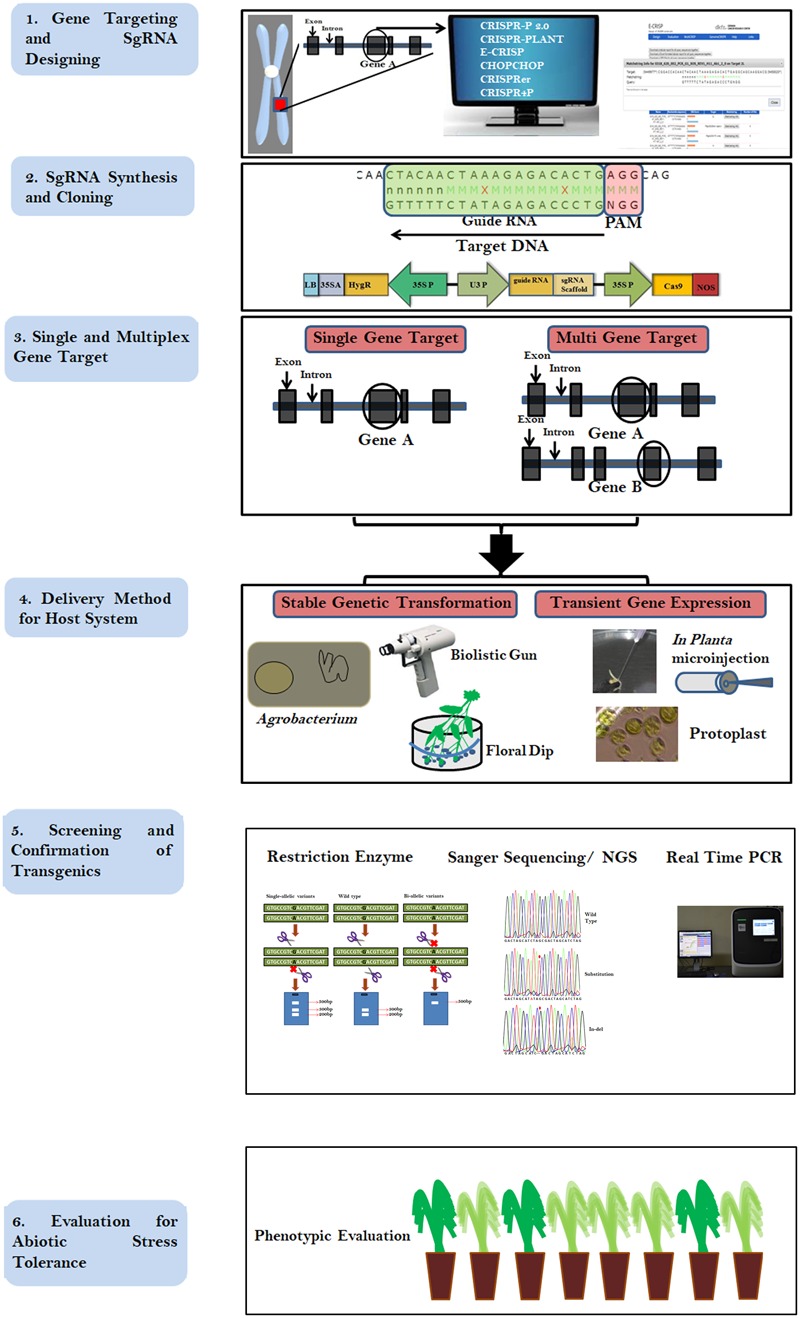
Flow chart describing the steps involved in CRISPR/Cas9 based genome editing. Step 1 describes the selection of gene and designing of gRNA, Step 2 describes the cloning of the gRNA in a suitable binary vector. Step 3 Shows the availability single and multiplex editing. Step 4 describes methods of transformation, Step 5 explains screening methods of edited crops and Step 6 demonstrates the evaluation and selection of the desirable transgene-free plant for the target trait.

**FIGURE 2:**
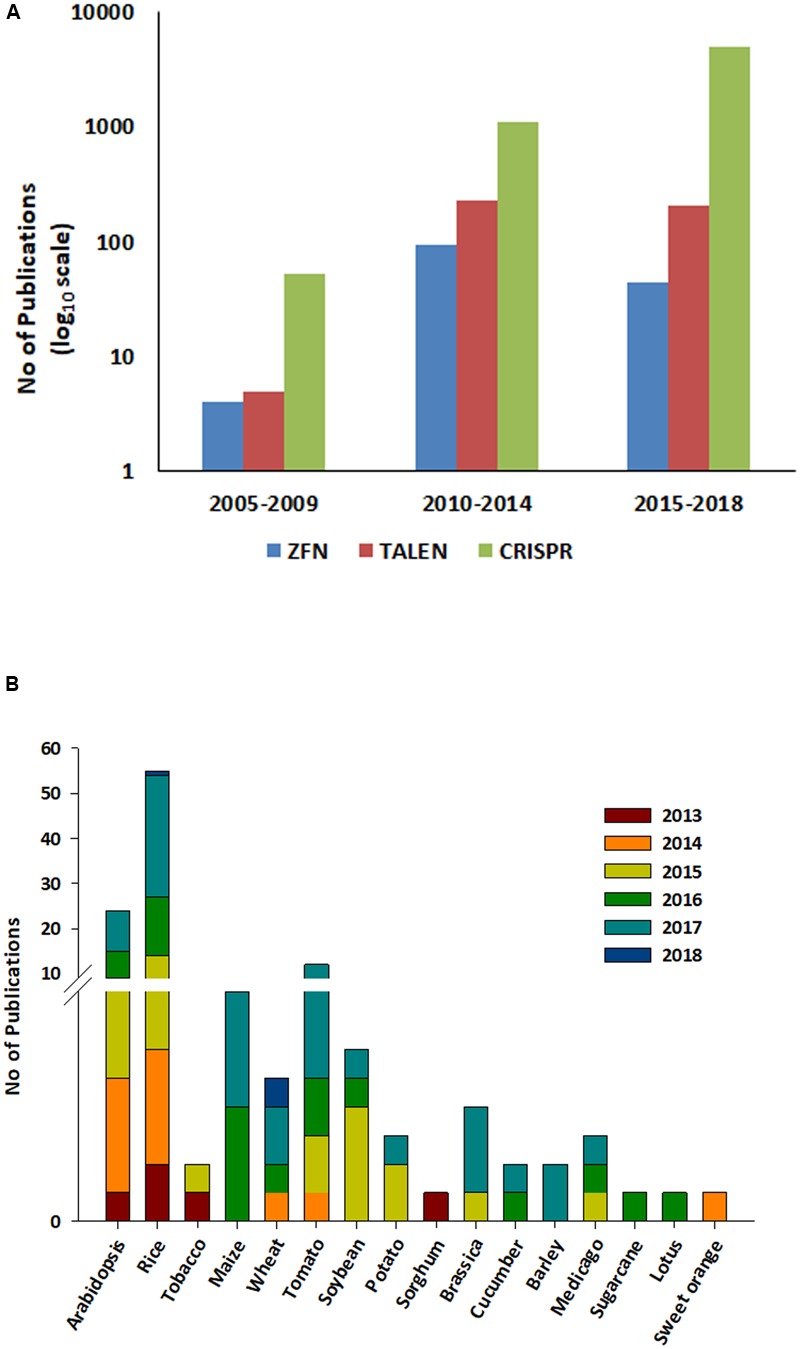
A research and review articles published on ZFN, TALEN, and CRISPR from 2005 to 2018. **(A)** ZFN, TALEN, and CRISPR were used as a search word in the title using the web of science search engine. Data was collected for three specified durations (i) 2005–2009, (ii) 2010–2014 and (iii) 2015–2018 (Feb 8th, 2018) (https://webofknowledge.com/). Each bar in the graph denotes each techniques and the color coding is described at the bottom of the figure. **(B)** Data on research articles published in plants during last 5 years (2013–2018). Data was collected using ‘CRISPR and crop name,’ e.g., ‘CRISPR rice’ in the title using the web of science 2013–2018 (https://webofknowledge.com/). Each bar denotes one year and the color coding is described at the top right of the figure.

The CRISPR cleavage methodology requires (i) a short synthetic gRNA sequence of 20 nucleotides that bind to the target DNA and (ii) Cas9 nuclease enzyme that cleaves 3–4 bases after the protospacer adjacent motif (PAM; generally 5′ NGG; Jinek et al., 2012). The Cas9 nuclease is composed of two domains, (a) RuvC-like domains and (b) a HNH domain, with each domain cutting one DNA strand. Following the development of the CRISPR cleavage methodology, it has been widely applied in plant and animal genome editing. Between 2010 and 2018, nearly 5000 articles have been published detailing the use of CRISPR^1^. Implementing a CRISPR project involves simple steps *viz.* (i) identifying the PAM sequence in the target gene, (ii) synthesizing a single gRNA (sgRNA), (iii) cloning the sgRNA into a suitable binary vector, (iv) introduction into host species/cell lines transformation followed by (v) screening and (vi) validation of edited lines (**Figure 1**). The simple steps involved in CRISPR/Cas9 mediated genome editing (CMGE) allows even a small laboratory with a fundamental plant transformation set up to carry out genome editing projects. CRISPR/Cas9 techniques have been used more extensively to edit plant genomes in the last half decade compared to ZFNs/TALENs and are reflective of its ease of use (**Figure 2A**). However, in plants, most editing has been demonstrated in model species such as *Arabidopsis*, rice and tobacco and only a few crop species have been researched using CRISPR technology (Jiang et al., 2013; **Figure 2B**).

## Improvements to CRISPR/Cas9 Editing Technology

**FIGURE 3:**
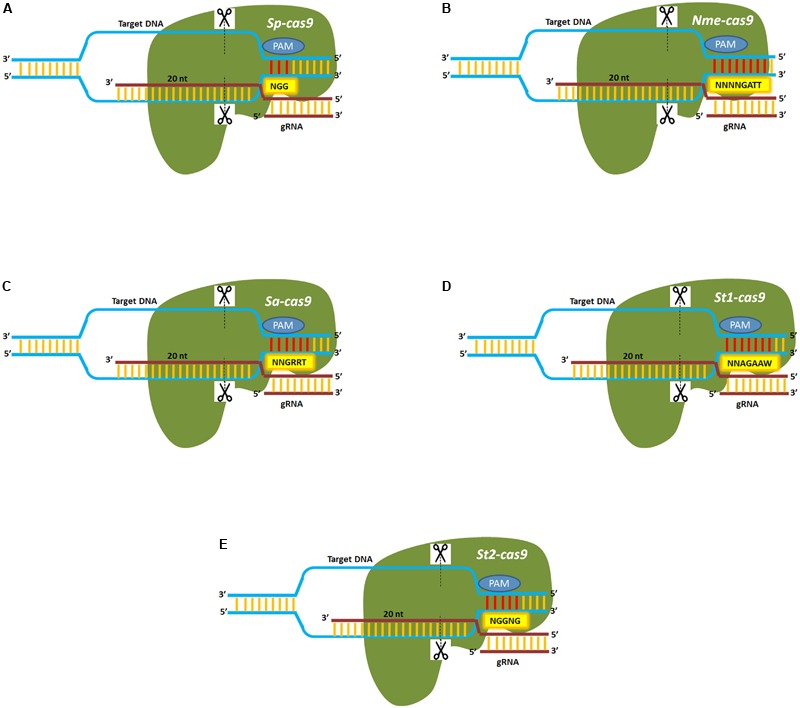
Cas9 orthologs from bacterial species show differences in their PAM repertoire. **(A)** *Sp-cas9* derived from *Streptococcus pyogenes* recognizes a three nucleotide PAM (5′-NGG) sequence. **(B)** *Nme-cas9* derived from *Neisseria meningitidis* recognizes an eight nucleotide PAM (5′-NNNNGATT) sequence. **(C)** *Sa-cas9* derived from *Staphylococcus aureus* recognizes a six nucleotide PAM (5′-NNGRRT) sequence. **(D)** *St1-cas9* derived from *Streptococcus thermophilus* recognizes a seven nucleotide PAM (5′-NNAGAAW) sequence. **(E)** *St2-cas9* derived from *Streptococcus thermophilus* recognizes a five nucleotide PAM (5′-NGGNG) sequence. Dotted lines indicate the site of the double-strand break.

One of the significant limitations of the CRISPR/Cas9 system, first derived from *Streptococcus pyogenes*, is the generation of significant off-target cleavage sites as a result of complexing of the gRNA with mismatched complementary target DNA within the genome. Thus, several modifications of the Cas9 enzyme have been developed to increase target specificity and reduce off-target cleavage and are listed in **Table 1**. An increase in the protospacer adjacent motif length is another strategy that is being used to minimize off-target cleavage. Cas9 proteins isolated from different bacterial species had unique and expanded PAM sequences that can aid in increasing on-target specificity and are shown in **Figure 3**. The CRISPR-Cas9 system derived from *Neisseria meningitidis* known as Nmecas9, recognizes an 8-mer PAM sequence (5′-NNNNGATT) that can improve target specificity and reduce potential off-target cleavage (Lee et al., 2016) while *Staphylococcus aureus*, Sacas9 recognizes a 6-mer PAM sequence (5′-NNGRRT; Ran et al., 2015). Two Cas9 cassettes obtained from *Streptococcus thermophilus* (st1cas9 and st3cas9) used to edit two human loci, *PRKDC* and *CARD11*, showed reduced off-target rates compared to the previously developed SpCas9 (Muller et al., 2016). Hu et al. (2018) demonstrated the modification of spcas9 (Cas9-VQR; Hu et al., 2016) which efficiently edit the target gene with 5′NGA PAM. Finally, several CRISPR/Cas9 orthologs have also been identified to improve target specificity (**Table 2**). CRISPR-CpfI is a class II, type V endonuclease developed from *Prevotella* and *Francisella* (Zetsche et al., 2017). In contrast to Cas9, CpfI requires a single RNA guided (crRNA) complex for cleavage and produces cohesive ends with 4–5 nucleotides 5′-overhangs. The CRISPR-cpfI system has been used successfully in both plant and animal systems and shown to have less or no off targets (**Table 3**). Besides cpf1, nearly 53 other CRISPR/Cas candidates have been characterized, among which the C2c2 nuclease isolated from *Leptotrichia shahii* is capable of dual nuclease activity and can target single-stranded RNA (Zaidi et al., 2017).

**Table 1:** List of Cas9 modifications and its applications.

Modification		Engineering	Application	Reference
SpCas9n (Cas9n)		Substitution of aspartite to alanine (D10A) in the RuvC domain	Allows knock in via HDR	Cong et al., 2013
Dead cas9 (dcas9)		Cas9 nuclease inactivation and double nicking using nickase	Nicking enhances specificity	Mali et al., 2013
FokI Cas9 (fCas9)		Inactivated Cas9 nuclease fused with FokI nuclease	Increased on target activity	Guilinger et al., 2014

**Table 2:** List of CRISPR/Cas9 orthologs.

System	gRNA		Source	Protein	PAM (5′–3′)	Reference	
CRISPR-cas9	tracrRNA+ crRNA		*Streptococcus pyogenes*	Cas9	NGG	Jinek et al., 2012	
CRISPR-cpf1	Single RNA		*Prevotella and Fracisella*	Cas1, Cas2, Cas4	YTN	Zetsche et al., 2017	
Ng-Ago	Single RNA		*Natronobacterium gregoryi*	Argonaute	Not required	Gao et al., 2016	

**Table 3:** Application of CRISPR-Cpf1 system in crops.

Crop		System	Source	Gene of interest	Trait	Reference
Rice		Fncpf1	*Francisella novicida*	*OsDL, OsALS*	Leaf morphology	Endo et al., 2016
Tobacco		Fncpf1	*Francisella novicida*	NtPDS, NtSTF1	Pigmentation, Leaf morphology	Endo et al., 2016
Rice		Lbcpf1	*Lachnospiraceae bacterium*	OsEPFL9	Stomatal development	Yin et al., 2017

## CRISPR/Cas9 Vectors for Gene Editing in Plants

As in other animal model systems, Cas9 and sgRNA expression within targeted cell is sufficient to modify plant genomes. Plant-specific RNA polymerase III promoters [AtU6 (*Arabidopsis*); TaU6 (wheat); OsU6 or OsU3 (rice)] are used to express Cas9 and gRNA in plant systems. There are several commercially available vectors for expressing Cas9 or Cas9 variants and gRNAs in plant systems. Addgene is a global, non-profit repository for plasmids which can currently make available more than 30 empty gRNA backbones in binary vectors^2^. The empty gRNA backbones have plant RNA polymerase III promoter and gRNA scaffolds to which a researcher can insert the gRNA of interest.

## CRISPR for Crop Improvement

**FIGURE 4:**
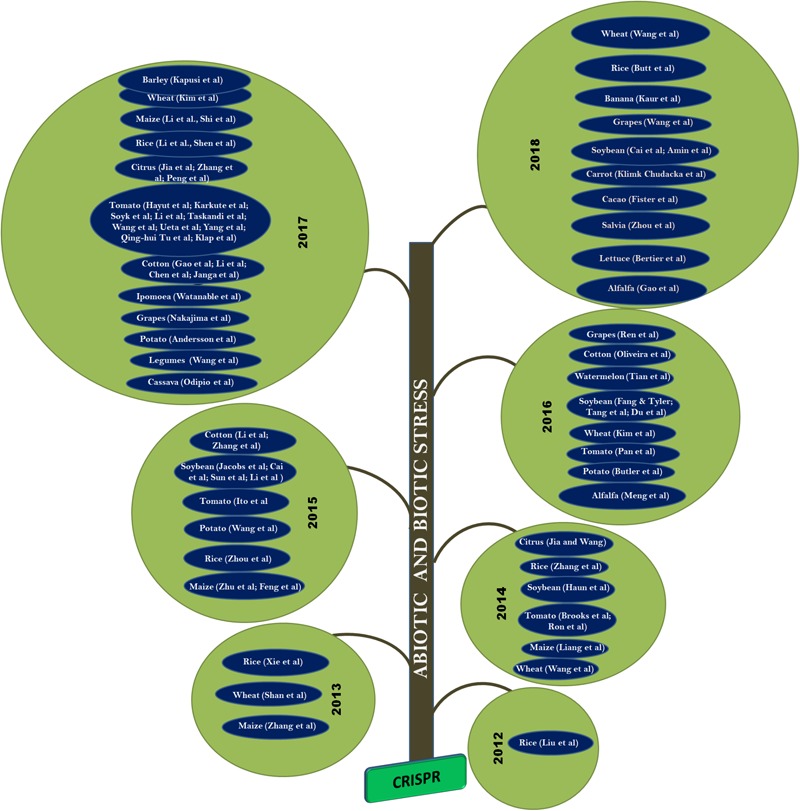
Application of CRISPR/Cas9 approach in plants: In 2013, CRISPR was demonstrated on rice, wheat, and maize. Whereas, in 2014, the technique was applied to tomato, soybean, and citrus. It was adopted in cotton and potato during 2015. Followed by watermelon, grapes, and alfalfa in 2016. CRIPSR/Cas was also applied to cassava, ipomoea, and legumes during 2017. Its is also applied to carrot, cacao, salvia, and lettuce during 2018 and many more crops yet to be reported.

CRISPR/Cas9 method of gene editing has been adopted in nearly 20 crop species so far (Ricroch et al., 2017) for various traits including yield improvement, biotic and abiotic stress management. Many of the published articles are considered as proof-of-concept studies as they describe the application of CRISPR/Cas9 system by knocking out specific reported genes playing an important role in abiotic or biotic stress tolerant mechanisms. Biotic stress imposed by pathogenic micro-organisms pose severe challenges in the development of disease-resistant crops and account for more than 42% of potential yield loss and contribute to 15% of global declines in food production (Oerke, 2005). CRISPR/Cas9-based genome editing has been utilized to increase crop disease resistance and also to improve tolerance to major abiotic stresses like drought and salinity (**Figure 4**). A survey of the use of CRISPR for genome editing in various crop species is presented below.

## Monocots

### Rice

Rice (*Oryza sativa*) is a major staple food crop for more than half of the world population and due to its small genome size, it is well studied and serves as a model crop for monocots. In the recent past, several studies have been demonstrated the application of CRISPR based genome editing approach in rice and few studies reported the utilization of genome editing for improving biotic and abiotic stresses for rice crop improvement (**Table 4**).

**Table 4:** Application of CRISPR based genome editing appraoch in plants for biotic, abiotic, and nutritional traits.

Crop	Method	Target gene	Stress/trait	Reference
Biotic Stress				
*A. thaliana*/ *N. benthamiana*	NHEJ	dsDNA of virus (A7, B7, and C3 regions)	Beet severe curly top virus resistance	Ji et al., 2015
*A. thaliana*	NHEJ	*eIF(iso)4E*	Turnip mosaic virus (TuMV) resistance	Pyott et al., 2016
*N. benthamiana*	NHEJ	BeYDV	Bean yellow dwarf virus (BeYDV) resistance	Baltes et al., 2015
*N. benthamiana*	NHEJ	ORFs and the IR sequence sDNA of virus	Tomato yellow leaf curl virus (TYLCV) and Merremia mosaic virus (MeMV)	Ali et al., 2015
Rice	NHEJ	*OsERF922* (ethylene responsive factor)	Blast Resistance	Wang F. et al., 2016
Rice (IR24)	NHEJ	*OsSWEET13*	Bacterial blight disease resistance	Zhou et al., 2015
Bread wheat	NHEJ	*TaMLO-A1, TaMLO-B1*, and *TaMLOD1*	Powdery mildew resistance	Wang et al., 2014
Cucumber	NHEJ	*eIF4E* (eukaryotic translation initiation factor 4E)		Chandrasekaran et al., 2016
			Cucumber vein yellowing virus (CVYV), Zucchini yellow mosaic virus (ZYMV), and Papaya ring spot mosaic virus type-W (PRSV-W)	
Abiotic stress		
Maize	HDR	*ARGOS8*	Increased grain yield under drought stress	Shi et al., 2017
Tomato	NHEJ	*SlMAPK3*	Drought tolerance	Wang et al., 2017
*A. thaliana*	NHEJ	*UGT79B2, UGT79B3*	Susceptibility to cold, salt, and drought stresses	
*A. thaliana*	HDR	*MIR169a*	Drought tolerance	Zhao et al., 2016
*A. thaliana*	NHEJ	*OST2* (OPEN STOMATA 2) (AHA1)	Increased stomatal closure in response to abscisic acid (ABA),	Osakabe et al., 2016
Rice	HDR, NHEJ	*OsPDS, OsMPK2, OsBADH2*	Involved in various abiotic stress tolerance	Shan et al., 2013
Rice	NHEJ	*OsMPK5*	Various abiotic stress tolerance and disease resistance	Xie and Yang, 2013
Rice	NHEJ, HDR	*OsMPK2, OsDEP1*	Yield under stress	Shan et al., 2014
Rice	NHEJ	*OsDERF1, OsPMS3, OsEPSPS, OsMSH1, OsMYB5*	Drought tolerance	Zhang et al., 2014
Rice	NHEJ	*OsAOX1a, OsAOX1b,OsAOX1c, OsBEL*	Various abiotic stress tolerance	Xu et al., 2015
Rice	NHEJ	*OsHAK-1*	Low cesium accumulation	Cordones et al., 2017
Rice	NHEJ	*OsPRX2*	Potassium deficiency tolerance	Mao et al., 2018
Nutritional and other Traits		
Rice	NHEJ	*25604 gRNA for 12802 genes*	Creating genome wide mutant library	Meng et al., 2017
Maize	NHEJ	*ZmIPK1A ZmIPK* and*ZmMRP4*	Phytic acid synthesis	Liang et al., 2014
Wheat	HDR	*TaVIT2*	Fe content	Connorton et al., 2017
Soybean	NHEJ	*GmPDS11* and *GmPDS18*	Carotenoid biosynthesis	Du et al., 2016
Tomato	NHEJ	*Rin*	Fruit ripening	Ito et al., 2015
Potato	HDR	*ALS1*	Herbicide resistance	Butler et al., 2016
Cassava	NHEJ	*MePDS*	Carotenoid biosynthesis	Odipio et al., 2017

#### Proof of Concept Studies

The rice genome shows an abundance of potential PAM (1 in 10 bp) sites (Xie and Yang, 2013). CRISPR technology can thus be potentially used to target any trait of interest in the rice genome in the near future. Shan et al. (2013) demonstrated sequence-specific CRISPR/Cas9 mediated genomic modification of three rice genes, phytoene desaturase (*OsPDS*), betaine aldehyde dehydrogenase (*OsBADH2*) and mitogen-activated protein kinase (*OsMPK2*) genes that are involved in controlling responses to various abiotic stress stimuli for the first time in any crop plant using both protoplast and particle bombarded rice calli systems. Nearly nine and seven percent of editing rates were observed for *OsPDS* and *OsBADH2*, respectively. Xie and Yang (2013) have demonstrated a RNA-guided genome editing approach by developing two vectors suitable for genome editing in rice, pRGE3 and pRGE6. *OsMPK5*, a negative regulator of biotic and abiotic stresses in rice was selected for targeted mutagenesis using three gRNAs and tested in rice protoplasts. A low level of off-targets was reported using a more precise gRNA design approach.

The efficiency of the CRISPR/Cas9 system in inducing targeted mutation and the heritability in mutant rice lines were evaluated for a list of genes including *OsDERF1, OsPMS3, OsEPSPS, OsMSH1, OsMYB5* (Zhang et al., 2014). A wide variation in mutation rates (21–66%) was observed in the in T_0_ generation for various genes with no or 1 bp off-target mutation and upto 11% of homozygous mutants were observed in the T_2_ generation. Targeted base editing of the herbicidal gene, *C287* in rice was made possible using activation-induced cytidine deaminase (Target-AID) method (Shimatani et al., 2017) in which dCas9 fused with cytidine deaminase was used for base editing without introduction of DSBs. Similarly, Zong et al. (2017) demonstrated a precise genome editing in rice, wheat and maize. Li et al. (2017a) demonstrated base editing of rice *OsPDS* and *OsSBEIIb* genes using BE3 base editor. BE3 base editor is an improved genome editing tool that combines nicked cas9 (ncas9- a D10 mutation in cas9), cytosine deaminase and the uracil glycosylase inhibitor (UGI) that inhibits base-excision repair. This study demonstrated the successful application of base editing in rice. Multiplex genome editing of a potentially unlimited number of genes is now made easy by CRISPR/Cas9 (Lowder et al., 2015) and has been recently demonstrated in rice and *Arabidopsis* (Zhang et al., 2016; Shen L. et al., 2017). Shen L. et al. (2017) have successfully edited eight agronomic genes using one binary vector for each genetic transformation in rice. The genes were ligated using the isocaudamer method involving intermediate vectors. The study reported reduced off targets and showed a cascade of sgRNAs might not affect the mutation rate of CRISPR/Cas9.

#### Functional Studies of Biotic and Abiotic Stress-Related Genes

A CRISPR/Cas9 targeted mutation in the ethylene responsive factor, *OsERF922* in rice, has been successfully established to increase resistance to blast disease caused by *Magnaporthe oryzae* (Liu et al., 2012). The expression of the disease susceptibility gene, *OsSWEET13* in rice is essential for infection by *Xanthomonas oryzae* pv. *Oryzae* to cause bacterial blight (Zhou et al., 2015). CRISPR/Cas9 technology has been used to develop two knockout mutants of *OsSWEET13* that target its promoter, leading to improved resistance to bacterial blight disease in *indica* rice, IR24. Plant annexins play a significant role in plant development and protection of plants from environmental stresses. The important role played by rice annexin gene (*OsAnn3*) under cold stress was examined in *OsAnn3* CRISPR knockouts (Shen C. et al., 2017). Survival of T_1_ mutant lines was found to decrease compared to wild-type plants under cold treatment.

Several vital traits such a yield and abiotic stress tolerance are controlled by two or more genes. In crop improvement programs, numerous studies have attempted to map these quantitative regions (quantitative trait loci – QTL) controlling agronomically important traits. Such identified QTL regions introgressed into elite lines for developing better performing varieties. However, this introgression is tedious if the QTLs are linked closely and introducing non-target regions into elite line may cause deleterious effects. CRISPR/Cas9 system can be a potent tool to introduce and study rare mutations in crop plants. The function of grain size (*GS3*) and grain number QTLs (*Gn1a*) in rice varieties were examined using a CRISPR based-QTL editing approach (Shen et al., 2018). The study showed that the same QTL can have highly varied and opposing effects in different backgrounds.

### Wheat

Wheat is an important cereal grain, grown worldwide as a staple food crop. Shan et al. (2014) successfully demonstrated the application of CRISPR/Cas9 strategy in wheat protoplasts for *TaMLO* gene (Mildew resistance locus O). The CRISPR *TaMLO* knockout was also shown to confer resistance to powdery mildew disease caused by *Blumeria graminis* f. sp. *Tritici* (*Btg*). Of the 72 T_0_ knockout MLO wheat homoeolog (*TaMLO-A*) transgenic lines analyzed for restriction enzyme digestion using T7 endonuclease I (T7E1), four lines were found to be edited for the restriction enzyme site (Wang et al., 2014). Efficient construct delivery methods can improve or increase the number of transgenic lines obtained. T-DNA based delivery systems are commonly used to introduce SSNs and the gRNA. However, DNA-virus based amplicons appear to lead to several fold increases in gene targeting efficiencies. Gil-Humanes et al. (2017) have utilized wheat geminiviral based DNA replicons [wheat dwarf virus (WDV)] for transient and straightforward expression of CRISPR/Cas9 cassettes, resulting in a 12-fold increase in the expression of endogenous ubiquitin gene in hexaploid wheat. High frequency of gene targeting using WDV-based DNA replicons will be a potential method in genome engineering of complex genomes in the future.

Kim et al. (2018) have reported CRISPR/Cas9 genome editing system in wheat protoplasts for two abiotic stress-related genes namely, wheat dehydration responsive element binding protein 2 (*TaDREB2*) and wheat ethylene responsive factor 3 (*TaERF3*). Nearly 70% of protoplasts were transfected successfully and expression of these edited genes were confirmed with the T7 endonuclease assay. Significant concerns about the application of CMGE in crops are transgene integration and off-target mutations. To overcome these issues, Liang et al. (2017) demonstrated an efficient method of genome editing using the biolistic delivery method of CRISPR/Cas9 ribonucleoproteins (RNPs). In general, CRISPR/Cas9 DNA will be integrated into the host genome and expressed stably whereas, the biolistic method of delivering RNPs will provide transient expression and degraded rapidly by which it drastically reduces off-targets. Two different genes (*TaGW2* and *TaGASR7*) in two varietal backgrounds were edited using CRISPR/Cas9 RNP complex in bread wheat. As this complex is degraded *in vivo*, it dramatically reduces off-target effects and no off-targets were found in the mutant bread wheat population. An extended protocol of RNP delivery has been made available by Liang et al. (2018). This DNA-free editing method avoids time consuming procedures such as backcross breeding for the removal of the transgene and allows to obtain transgene-free plants at T_0_. However, this method has limitations including like low efficiency rates compared to CRISPR/Cas9 DNA binary delivery systems as the expression is transient and also requires laborious mutant screening with no marker selection applied during the development. If these limitations can be overcome, the RNP method will be an efficient approach to achieve CRISPR/Cas9 based genome editing in crop species, especially perennial crops. CRISPR/Cas9 based multiplexed genome editing has been demonstrated for model crops to edit many important agronomic traits simultaneously. Recently Wang W. et al. (2018) reported the frequency of mutations and heritability generated through multiplexed genome editing in hexaploidy wheat. Three wheat genes, *TaGW2* (a negative regulator of grain traits), *TaLpx-1* (lipoxygenase, which provides resistance to *Fusarium graminearum*) and TaMLO (loss of function, confers resistance to powdery mildew resistance) were targeted in this study using three gRNAs combined in tRNA spaced polycistronic cassette under the transcriptional control of a single TaU3 promoter. Editing efficiency was tested in wheat protoplasts and the DNA was evaluated for editing/mutations by next-generation sequencing followed by *Agrobacterium*-mediated transformation and mutant screening. Statistical and phenotypic analysis was carried out in successive generations *viz.*, T_0_, T_1_, T_2_, and T_3_ and editing efficiencies were observed for the three homeologous copies. This study showed that transgenerational gene editing activity can serve as the source of novel variation in the progeny of CRISPR-Cas9-expressing plants. This approach will be an efficient method for multiplex genome editing in complex crops like polyploid crop species.

### Maize

Maize (*Zea mays*) is a major cereal crop and phytic acid constitutes more than 70% of the maize seed. It is believed to be anti-nutritional as it is not digested by monogastric animals and is also an environmental pollutant. Liang et al. (2014) have reported targeted knock out of genes involved in phytic acid synthesis (*ZmIPK1A, ZmIPK*, and *ZmMRP4*) in *Z. mays*. Similarly, Zhu et al. (2016) demonstrated gene editing of phytoene synthase gene (*PSY1*) using maize U6 snRNA promoter. *PSY1* is involved in carotenoid biosynthesis and its mutant (*psy1*) results in white kernels and albino seedlings. Among fifty two T_0_ lines obtained by *Agrobacterium*-mediated transformation, seven lines were reported to carry the *psy1* knockout trait and all seven lines were deep sequenced to understand the type of variation and to evaluate the mutation efficiency. The results showed that no off-target sites were edited and stable *psy1* mutants were obtained. Feng et al. (2016) have demonstrated the utility of the CRISPR/Cas9 system in maize by targeting the albino marker gene, *Zmzb7* in a protoplast system. Knockout of *Zmzb7* results in albino plant, with the sgRNA designed to target a region in the eighth exon of *Zmzb7* and maize U3 promoter was used for expression. Following *Agrobacterium*-mediated transformation of maize embryos, T_0_ lines were found to show a 31% mutation efficiency.

Gene editing tools that can effect multiple gene knockouts are of immense importance to accelerate and achieve efficient crop breeding. For the first time, multiplex genome editing in maize was demonstrated by Qi et al. (2016) using a tRNA-RNA processing system. A multiplex editing vector can incorporate a cluster of gRNAs separated by spacers in a polycistron, producing multiple gRNAs from one primary transcript. The study targeted three transcription factor genes (*MADS, MYBR*, and *AP2*) for simplex editing and three other genes (*RPL, PPR*, and *IncRNA*) for multiplex editing. Increased editing efficiency (upto 100%) was observed for t-RNA processing based multiplex editing. Current high yielding maize varieties are the result of hybrid maize seed production and the production of hybrid maize requires sterilization to avoid self-fertilization. Maize *thermosensitive genic male-sterile 5* (*ZmTMS5*), known to cause male sterility was targeted for genome editing by CRISPR/Cas9 approach (Li et al., 2017b). Three gRNAs were used to knockout the gene, with one sgRNA targeting the first exon and the other two sgRNAs targeting the second exon. Mutation efficiency was examined in maize protoplasts using PCR/restriction enzyme assays. Analysis of mutational efficiency revealed that the sgRNA targeting the first exon had no off targets whereas the other two sgRNAs had off-targets in the maize genome. The *AUXIN REGULATED GENE INVOLVED IN ORGAN SIZE* (*ARGOS*) gene family are negative regulators of the ethylene response and modulate ethylene signal transduction. Overexpression of *ARGOS* genes in transgenic maize plants enhances drought tolerance and identification of new allelic variants would be of immense importance in maize breeding programs. Shi et al. (2017) utilized CRISPR/Cas9 genome editing to create new allelic variants of *ARGOS8*. Two genome edited variants (*ARGOS8-v1* and *ARGOS8-v2*) were used for the production of hybrids and evaluated in the field in multi-location trials. Improved yield under stress observed for the variant hybrid than the wild-type. This study demonstrates the use of CRISPR/Cas9 genome editing method for creating new variants and their application in crop improvement.

## Genome Editing in Other Monocots

Apart from model crops, CRISPR/Cas9 genome editing approach has been applied to other monocot crop species for improving essential traits. Kapusi et al. (2017) demonstrated CRISPR/Cas9 based knock out in barley for the endo-*N*-acetyl-b-D-glucosaminidase (*ENGase*) gene. A set of five gRNAs were designed to knockout ENGase using both particle bombardment and *Agrobacterium*-mediated transformation. Genotyping of T_0_ and T_1_ mutant barley lines showed 78% of mutational efficiency. Such knockout plants will be useful for studying the function of genes in functional genetics. Recently, Kaur et al. (2018) demonstrated CRISPR/Cas9 modification in banana cv. Rasthali of the *phytoene desaturase* (*RAS-PDS*) gene that is involved in the carotenoid biosynthesic pathway. Knock out *RAS-PDS* in banana using CRISPR produced thirteen mutant lines that were evaluated for carotenoid and chlorophyll content. This study paves the way for the application of CRISPR/Cas9 gene editing in banana and will help accelarate further research in the development of banana plants with desirable traits.

## Dicots

### Arabidopsis

CRISPR/Cas9 based target genome editing was demonstrated for the first time by Feng et al. (2013) in *Arabidopsis*. Three phenology related *Arabidopsis* genes, *brassinosteroid insensitive1* (*BRI1), jasmonate-zim-domain protein 1* (*JAZ1*) and *gibberellic acid insensitive* (*GAI*) were edited using floral dip method and genotyped using Restriction Fragment Length Polymorphism. Further sequencing confirmed the high efficiency of mutation (26–84%). In another study, Mao et al. (2013) demonstrated CRISPR/Cas9 genome editing of albinism related genes, *magnesium-chelatase subunit I* (*CHLI1*) and *CHLI2 in Arabidopsis*, with mutant plants being screened by Amplified Fragment Length Polymorphism. They demonstrated the importance of the new genome editing tool to effect gene correction and deletion of large genomic fragments in a plant genome. To study the efficiency, heritability, specificity, and pattern of modified genes using CRISPR/Cas9 genome editing, Feng et al. (2014) monitored the flow of seven genes, targeting 12 loci in *Arabidopsis* over succeeding generations. Predominately, 1-bp insertions and small deletions were observed among the edited lines with high mutation rates (around 58–79%) in T_1_ to T_3_ generations. Homozygous mutants were passed to the next generation without any modifications and no off-targets were observed. This study demonstrated, the generation of the heritable alterations through CRISPR/Cas9 genome editing in plants. Multiplex CRISPR/Cas9 was also demonstrated to target many regions of the same gene by Lowder et al. (2017). Simultaneous targeting of three different regions on the TRANSPARENT TESTA_4_ (TT_4_) gene in *Arabidopsis thaliana* using multiplex CRISPR/Cas9 was made possible through Golden Gate cloning and Multisite Gateway LR recombination methods.

CRISPR/Cas9 genome editing of five *A. thaliana* genes: *PDS3 (PHYTOENE DESATURASE), AtFLS2 (FLAGELLIN SENSITIVE 2), CYCD3 (CYCLIN D-TYPE 3), RACK1 (RECEPTOR FOR ACTIVATED C KINASE 1-AtRACK1b* and *AtRACK1c)* was examined in protoplasts (Li et al., 2013). There was variability in mutational efficiencies that could be attributed to sgRNA binding strength or chromatin structure. This study also demonstrated the efficiency of multiple gRNAs in bringing about gene editing effects. With floral dip being the preferred mode of transformation in *Arabidopsis*, there have been attempts to obtain germline mutants by targeting germline tissues through the use of tissue-specific promoters and terminators (Wang et al., 2015). Mao et al. (2016) have developed a germ-line-specific Cas9 system (GSC) for *Arabidopsis* by utilizing 5′ regulatory sequences of three genes (*SPOROCYTELESS, DD45* and tomato *LAT52*) from *Arabidopsis* that target floral organs to drive Cas9 expression. A significant increase in rates of heritable mutations, reduction in the proportion of chimeras and increase in mutation diversity in the T_2_ generation was achieved, thus providing a specific CRISPR/Cas9 system for genetic screening of lethal or other desired mutations in *Arabidopsis*. Turnip mosaic virus (TuMV) is a devastating viral disease caused in field-grown vegetable crops. Loss-of-function mutations in components of the eukaryotic translation initiation factor, eIF4F translation complex are associated with stable resistance to several potyviruses. CRISPR/Cas9 genome editing was adopted to introduce sequence-specific deleterious pointmutations at the eIF(iso)4E locus in *Arabidopsis* to successfully engineer complete resistance to TuMV (Pyott et al., 2016). Zhang T. et al. (2018) utilized FnCas9 for establishing a CRISPR/Cas9 based interference (CRISPRi) system to confer TuMV resistance. LeBlanc et al. (2018) established a higher frequency of CRISPR-induced mutations in *Arabidopsis*, by approximately 5-fold in somatic tissues and up to 100-fold in the germline due to heat stress (37°C), relative to plants grown continuously at the standard temperature (22°C).

### Cotton

In addition to being a fiber crop, cotton is also a good source for biofuel production as cotton seeds contain significant oil reserves (Oliveira et al., 2016). With the release of the genome sequence of *Gossypium hirsutum* (Li F. et al., 2015), it is now possible to utilize CRISPR tools to achieve precise DNA modifications. Janga et al. (2017) first reported the targeted gene editing in cotton using CRISPR/Cas9 system. Green fluorescent protein (GFP) integrated transgenic cotton was targeted for genome editing with three target sites in the GFP sequence as a visual marker for phenotypic characterization. Of the nine T_0_ plantlets examined, for knockout by gRNA2, showed homozygous changes while seven others showed bi-allelic indels. The ability to introduce DSB at a precise target site has been further extended to create a precise nucleotide substitution or insertion of the desired DNA sequence through homology-dependent repair (Janga et al., 2017). Chen et al. (2017) examined the efficiency of genome editing in cotton by targeting two guide RNAs, one each for Cloroplastos alterados 1 (GhCLA1) and vacuolar H+-pyrophosphatase (GhVP) genes. In transformed plants, most of the mutations were nucleotide deletions, with mutational efficiencies of 47.6–81.8%. Cultivated cotton is an allotetraploid and posses significant challenges in developing site-specific DNA changes. Gao et al. (2017) established the efficacy of the CRISPR/Cas9 system in being able to produce mutations in homeologous cotton genes and also demonstrated multiple gene targeting can be achieved in cotton with the simultaneous expression of several sgRNAs. Li et al. (2017c) have demonstrated CRISPR/Cas9-induced specific truncation events in the cotton fiber development controlling *GhMYB25* homoeologous genes (*GhMYB25-like A* and *GhMYB25-like D*) in transgenic cotton through PCR amplification and sequencing analysis. Lately, resistance to *Verticillium dahliae* infestation was reported through gene editing of *Gh14-3-3d* gene. The resulting transgene-clean plants showed a high resistance and can be used as a germplasm to breed disease-resistant cotton cultivars (Zhang Z. et al., 2018).

### Soybean

Soybean (*Glycine max*), one of the most important seed oil crop with high seed protein content. The seed also contains a variety of physiologically active substances that are beneficial to humans. Cai et al. (2015) first successfully achieved CRISPR/Cas9-mediated genome editing in soybean using a single sgRNA for a transgene (*bar*) and six sgRNAs that targeted different sites of two endogenous soybean genes (*GmFEI2* and *GmSHR*) and examined efficacy of the sgRNAs in a hairy root system. Targeted mutagenesis of two genomic sites in soybean chromosome 4 (DD20 and DD43) resulted in small deletions and insertions (Li Z. et al., 2015). Targeted gene integrations through HDR were detected by border-specific polymerase chain reaction analysis at callus stage. Soybean *GmU6-16-1* promoter was found to be more efficient in simultaneous editing of multiple homoeoalleles relative to the *Arabidopsis At*U6*-26* promoter (Du et al., 2016). The role of a dominant nodulation restriction gene in soybean, *Rj4*, that inhibits nodulation by many strains of *Bradyrhizobium elkanii* was shown through both complementation and CRISPR/Cas9-mediated gene knockout experiments (Tang et al., 2016). CRISPR was used to disrupt the pathogen virulence gene (Avr4/6) in *Phytophthora sojae* (Fang and Tyler, 2016). Homologous gene replacement of Avr4/6 by a marker gene (NPT II) stimulated by the CRISPR/Cas9 system emphasized the contribution made by the virulence gene in recognition of the pathogen by plants containing the soybean R gene loci, *Rps4* and *Rps6*. CRISPR knockout of the soybean flowering time gene, *GmFT2*, was stably heritable in the subsequent T_2_ generation, with homozygous *GmFT2a* mutants exhibiting late flowering under both long-day and short-day conditions (Cai et al., 2018).

### Tomato

Tomato (*Solanum lycopersicum* L.), is an economically important crop that is an ideal candidate for testing CRISPR/Cas9 gene editing, because of the availability of efficient transformation methodologies, functional genomic characterization and substantial background on quality improvement (Pan et al., 2016). Brooks et al. (2014) reported efficient CMGE of the tomato *ARGONAUTE* gene, *SlAGO7*, that could be easily distinguished phenotypically as mutants produced first leaflets without petioles. CRISPR mediated knockout of the *SHORT-ROOT* (*SHR*) gene in tomato hairy roots suggested conservation of gene function between *Arabidopsis* and tomato and also showed that *SHR* in tomato regulates expression of the transcription factor gene *SCARECROW* (*SCR*) and root length (Ron et al., 2014). Regulation of ripening is one of the most critical concerns in the study of fleshy fruit species. Ripening inhibitor (*RIN*), is a MADS Box transcription factor that is a master regulator controlling tomato fruit ripening. RIN-protein-defective mutants generated by CMGE produced incompletely ripening fruits confirming the important role of RIN in ripening (Ito et al., 2015). The role of tomato *lncRNA1459* in fruit ripening was confirmed by CMGE; *lncRNA1459* mutants showed repression of fruit ripening a well as inhibition of ethylene and carotenoid biosynthesis (Li et al., 2018a). Similarly, knocking out the RNA recognition motif-containing gene, *SlORRM4* delays tomato fruit ripening by lowering respiratory rate and ethylene production (Yang et al., 2017). HDR-mediated replacement of the dominant *ALC* (Alcobaca) gene with the recessive *alcobaca (alc)* increased shelf life of T1 homozygous tomato (Yu et al., 2017). Parthenocarpic tomato plants generated by introducing somatic mutations in the parthenocarpy related gene, *SlIAA9*, using CRISPR show morphological changes in leaf shape and seedless fruits (Ueta et al., 2017). CMGE in the tomato flowering repressor, SP5G, improves inflorescence architecture and fruit yield in tomato (Soyk et al., 2017). The literature survey above on improving fruit traits in tomato underscores the importance of applying CRISPR methodologies creatively in the background of basic biological trait information to obtain desired phenotypes or crop traits.

Mitogen-activated protein kinases (MAPKs) are important signaling molecules that respond to drought stress in tomato by safeguarding the cell membrane from oxidative damage and regulating the transcription of genes involved in drought stress. *Slmapk3* mutants generated through CMGE are more sensitive to drought stress and show more severe wilting symptoms (Wang et al., 2017). Impairment of the *POWDERY MILDEW RESISTANT4* (*PMR4*) ortholog, *SlPMR4* in tomato, encoding a callose synthase, confers resistance against the oomycete pathogen, *Oidium neolycopersici* (Eleni Koseoglou, 2017, Thesis). Using a multiplex CRISPR system targeting five key genes in the γ-aminobutyric acid (GABA) shunt pathway in tomatoes, 53 genome-edited plants were obtained following single plant transformation, including single to quadruple mutants. GABA accumulation in both the leaves and fruits of genomically edited lines was significantly enhanced, with GABA content in leaves of quadruple mutants being 19-fold higher than in wild-type plants (Li et al., 2018b). CMGE was also used to modify tomato *Phytochrome interacting factor* (*SIPIF4*) and *phytoene desaturase* (*SIPDS*) genes generating stable and heritable modifications, with clear albino phenotypes observed for the *psd* mutants (Pan et al., 2016). Hayut et al. (2017) have shown that CMGE can be used for precise reshuffling of chromosomal segments between homologous chromosomes in somatic cells of tomato using a visual marker gene in tomato PSY1. Somatic HR can be used for allelic replacement and has implications for shortening the time period of crop generation by allowing wild desired loci to be transferred to the crop vis-a-vis conventional back-crossing and associated problems with linkage drag.

### Potato

Potato is an important food crop for world food security, and with climate change, it is essential to breed potato to adapt as well as identify breeding material that can be used to extend the region within which it is cultivated. Potato starch quality is important in many of its food applications and an important area of research. The waxy genotype was developed in hexaploid potato by mutating granule-bound starch synthase (GBSS) gene using CMGE. Characterization of starch in genome-edited lines revealed only the presence of amylopectin, with a complete lack of amylose, confirming the knock-out of all four alleles of GBSS (Andersson et al., 2017). Similarly, multi-allelic mutagenesis has been achieved in potato by mutating ACETOLACTATE SYNTHASE1 (StALS1) (Butler et al., 2016).

### Citrus

Citrus is an economically important fruit crop. Xcc-facilitated agroinfiltration of SpCas9/sgRNA and SaCas9/sgRNA has been reported in sweet orange and *Citrus paradisi*, respectively, both targeting the *Phytoene desaturase* gene, *CsPDS* and *CpPDS* (Jia and Wang, 2014; Jia et al., 2017a). Improvement of citrus canker resistance has been made possible through targeted modification of the 5′ regulatory region of the *LATERAL ORGAN BOUNDARIES* (*CsLOB1*) gene. *CsLOB1* is the susceptibility gene for citrus canker and plays a critical role in promoting pathogen growth and erumpent pustule formation. Different alleles of *CsLOB1* contain the effector-binding element (EBEPthA4). Enhanced resistance to citrus canker is observed in promoter disrupted *CsLOB1* that targets the effector binding element. Deletion of the entire EBEPthA4 sequence from both *CsLOB1* alleles provided a high degree of resistance. Promoter editing of *CsLOB1* alone was sufficient to enhance citrus canker resistance in Wanjincheng orange (Peng et al., 2017). Mutation of the coding region of both alleles of the susceptibility gene *CsLOB1* generated citrus canker-resistant in Duncan grapefruit (Jia et al., 2017b). Rapid and efficient genome editing of citrus was reported using the *Arabidopsis* YAO promoter targeting the *PDS* gene, suggesting that *Arabidopsis* YAO promoter can drive Cas9 expression for efficient gene editing during early stages of shoot regeneration in citrus (Zhang et al., 2017).

### Grape

Grape is an economically valuable fruit, with breeders targeting numerous fruit quality traits such as aroma, disease and abiotic stress resistance, fruit size and skin color. Ren et al. (2016), successfully demonstrated targeted genome editing of *L-idonate dehydrogenase* gene (*IdnDH*) in ‘Chardonnay’ suspension cells and regenerated grape plantlets. No off-target mutations were detected in the tested putative off-target sites, suggesting high specificity of the CRISPR/Cas9 system in grape genome editing. Majority of the detected mutations in the transgenic cell mass involved 1-bp insertions or followed by 1- to 3-nucleotide deletions. Targeted mutagenesis of grape *phytoene desaturase* (*VvPDS*) resulted in albino leaves. The ratio of mutated cells was higher in older leaves, attributed to either increased incidence of DSBs or impaired repair mechanisms in older leaves (Nakajima et al., 2017). Wang Y. et al. (2016) have identified five types of CRISPR/Cas9 target sites in the widely cultivated grape species *Vitis vinifera* for potential genome editing. Editing using purified CRISPR/Cas9 ribonucleoproteins (RNPs) as delivery particles in grape protoplasts has been shown to be effective against the powdery mildew susceptibility gene, *MLO-*7 (Malnoy et al., 2016). Targeted mutagenesis of *VvWRKY52*, a transcription factor gene has elucidated its role in biotic stress responses. In addition, knockout of *VvWRKY52* in grape increased disease resistance to fungal infection (*Botrytis cinerea*) (Wang X. et al., 2018).

## Genome Editing in Other Dicots

CRISPR/Cas9 is a transformative tool to bring about targeted genetic alterations. In plants, high mutation efficiencies have reported in primary transformants following gene editing. However, many of the mutations analyzed are somatic and therefore not heritable. Knockout of the *9-cis-EPOXYCAROTENOID DIOXYGENASE4* (*NCED4*) gene (coding for the first step in abscisic acid biosynthesis) in lettuce (*Lactuca sativa*) cvs. Salinas and Cobham Green, increases seed germination at high temperatures with seeds of both cultivars capable of >70% germination efficiencies at 37°C. Knockouts of *NCED4* provide a whole-plant selectable phenotype that has minimal pleiotropic consequences. Targeting *NCED4* in a co-editing strategy could, therefore, be used to enrich for germline-edited events by merely germinating seeds at high temperature. Germination thermotolerance due to inactivation of *NCED4* provides a useful whole-plant selectable phenotype of pleiotropic effects on growth or stress tolerance (Bertier et al., 2018). The *Non-Expressor of Pathogenesis-Related PR3* gene from cocoa (*TcNPR3*) is a suppressor of defense responses and editing it in leaves of cocoa confers an increased resistance to infection with the cacao pathogen *Phytophthora tropicalis* and elevated expression of downstream defense genes (Fister et al., 2018). Efficient carrot genome editing of the anthocyanin biosynthetic pathway gene *flavanone-3-hydroxylase* (*F3H*) in a model purple-colored callus was used as a visual marker to identify successfully edited transformation events (Klimek-Chodacka et al., 2018).

Targeted mutagenesis of *squamosa promoter binding protein-like 9* (*SPL9*) gene in *Medicago sativa* (alfalfa), a model legumes crop was demonstrated and analyzed in a high-throughput manner using droplet digital PCR (ddPCR) and lines showing high mutation rates by restriction enzyme digestion/PCR amplification and sequencing. Overall efficiency of editing in the polyploid alfalfa genome was lower compared to other less-complex plant genomes (Gao et al., 2018). Meng et al. (2017) demonstrated the disruption of *phytoene desaturase* (*MtPDS*) gene in *Medicago truncatula*. Chandrasekaran et al. (2016) have utilized CRISPR/Cas9 genome editing system to develop virus resistance in cucumber (*Cucumis sativus*). Targeted mutation of the recessive gene *eukaryotic translation initiation factor 4E*(*eIF4E*) was found to confer immunity toward Cucumber vein yellowing virus (CVYV), Zucchini yellow mosaic virus (ZYMV), and Papaya ringspot mosaic virus type-W (PRSV-W). Transgenic watermelon plants harboring ClPDS mutation sand showed clear or mosaic albino phenotype, indicating that CMGE is technically 100% efficient in developing transgenic watermelon lines (Tian et al., 2017).

CRISPR/Cas9 mediated genome editing is also being applied for the improvement of horticulturally important crops such as vegetable and fruit crops for enhancing the shelf life, yield and disease resistance. Increasing data availability through whole genome sequencing and transcriptome sequencing of important horticultural crops will enhance the application of CMGE for crop improvement (Karkute et al., 2017). A recent review by Sattar et al. (2017) describes the possibilities and challenges of CMGE in date palm which is an important fruit crop. Successful knockout of *carotenoid cleavage dioxygenase4* (*InCCD4*) in the white-flowered *Ipomoea nil*, cv. AK77 caused the white petals to turn pale yellow, with a 20-fold increase in the total carotenoid content in petals of *ccd4* mutant plants. It suggested that in the petals of *I. nil*, in addition to low carotenogenic gene expression, carotenoid degradation contributes to low carotenoid content (Watanabe et al., 2018). Wang L. et al. (2016) reported efficient inactivation of a symbiotic nitrogen fixation related gene, *SYMRK* (symbiosis receptor-like kinase) in *Lotus japonicus*. Targeted genome editing has been applied to knockout the *rosmarinic acid synthase gene* (*SmRAS*) in the Chinese medicinal herb *Salvia miltiorrhiza*. Subsequently, expression and metabolomic analysis showed that the levels of phenolic acids, including rosmarinic acid (RA) and lithospermic acid B were reduced. *SmRAS* expression levels decreased in the successfully edited hairy root mutant lines (Zhou et al., 2018).

Wang Z. et al. (2018) successfully produced albino kiwifruit plantlets using two editing strategies that targeted the phytoene desaturase gene: the polycistronic tRNA-sgRNA cassette (PTG) (PTG/Cas9) and the traditional CRISPR (CRISPR/Cas9) expression cassette. The authors concluded that mutagenesis frequency of the PTG/Cas9 system was 10-fold higher than that of the CRISPR/Cas9. Recently, *Arabidopsis* U6-26 promotor was used for the successful expression of sgRNA in date palm (Li et al., 2018c). This study demonstrated multiplex expression of a sgRNA to target five rapeseed SPL3 homologous gene copies and reported 96–100% mutagenesis which were evaluated using polyacrylamide gel electrophoresis (PAGE)-based screening approach. CMGE technology thus has enormous potential in helping unlock a wealth of information about biosynthetic gene pathways in both dicot and monocot species that could translate into crop improvement.

## Regulatory Concerns for the Crops Developed Using Genome Editing Tools

New breeding technologies like ZFNs, TALENs, and CRISPR does not fall under the definition of a GMO under regulatory regimes in many countries. The United States Department of Agriculture (USDA) has stated stated that CRISPR/Cas9 edited crops can be cultivated and sold free from regulatory monitoring (Waltz, 2018). This can save several million dollars on getting regulations of GMO crops for the field test and data collections. In addition, it also reduces time as it usually takes several years to release a GMO crop. It also will remove the uncertainty of consuming GMO crops among the public. To date, there are five crops edited with CRISPR/Cas9 approach in the pipeline that USDA has declared not to regulate including a white button mushroom (*Agaricus bisporus*); resistance to browning was developed using CRISPR/Cas9 by knocking out a gene polyphenol oxidase (PPO) (Waltz, 2016). Similarly, waxy corn (*Z. mays*) with enriched amylopectin has been developed by inactivating an endogenous waxy gene *Wx1* and has also been exempted from GMO regulations. Green bristlegrass (*Setaria viridis*) with delayed flowering time achieved by deactivating the *S. viridis* homolog of the *Z. mays ID1* gene, Yield10 Bioscience edited camelina for increased oil content and drought tolerant soybean (*Glycine max*) edited for *Drb2a* and *Drb2b* genes will also not be subject to regulatory evaluation.

## Conclusion

New breeding techniques provide scientists the ability to precisely and quickly insert the desired traits than conventional breeding. CRISPR/Cas9 based genome editing is a fundamental breakthrough technique. Application of genome editing tools in crop improvement to enhance yield, nutritional value, disease resistance and other traits will be a prominent areas of work in the future. In the last 5 years, it is being applied vigorously in many plant systems for functional studies and combating biotic and abiotic stresses as well as to improve other important agronomic traits. Though several modifications to this technology have to lead to increasing on-target efficiency, most work carried is preliminary and needs further improvement. Nevertheless, CRISPR/Cas9 based genome editing will gain popularity and be an essential technique to obtain ‘suitably edited’ plants that will help achieve the zero hunger goal and maintain feed the growing human population.

## Author Contributions

DJ, KR, GS, and SJ wrote the manuscript. DJ and GV designed and revised the manuscript.

## Conflict of Interest Statement

The authors declare that the research was conducted in the absence of any commercial or financial relationships that could be construed as a potential conflict of interest.

## Footnotes

1 https://webofknowledge.com/

2 https://www.addgene.org/
